# Hyperglycemia disrupted the integrity of the blood‐brain barrier following diffuse axonal injury through the sEH/NF‐κB pathway

**DOI:** 10.1002/iid3.1105

**Published:** 2023-12-06

**Authors:** Xing Wei, Zhiguo Xing, Tingqin Huang, Ming Zhang, Jinning Song, Yonglin Zhao

**Affiliations:** ^1^ Department of Gynaecology and Obstetrics The Second Affiliated Hospital of Xi'an Jiaotong University Xi'an China; ^2^ Department of Neurosurgery The First Affiliated Hospital of Xi'an Jiaotong University Xi'an China; ^3^ Department of Neurosurgery The Second Affiliated Hospital of Xi'an Jiaotong University Xi'an China; ^4^ Department of Oncology The Second Affiliated Hospital of Xi'an Jiaotong University Xi'an China

**Keywords:** diffuse axonal injury, hyperglycemia, nuclear transcription factor kappa B, soluble epoxide hydrolase

## Abstract

**Objectives:**

We aimed to investigate the role of soluble epoxide hydrolase for hyperglycemia induced‐disruption of blood‐brain barrier (BBB) integrity after diffuse axonal injury (DAI).

**Methods:**

Rat DAI hyperglycemia model was established by a lateral head rotation device and intraperitoneal injection of 50% glucose. Glial fibrillary acidic protein, ionized calcium‐binding adapter molecule‐1, β‐amyloid precursor protein, neurofilament light chain, and neurofilament heavy chain was detected by immunohistochemistry. Cell apoptosis was examined by terminal deoxynucleotidyl transferase nick‐end labeling (TUNEL) assay. The permeability of blood‐brain barrier (BBB) was assessed by expression of tight junction proteins, leakage of Evans blue and brain water content. The soluble epoxide hydrolase (sEH) pathway was inhibited by 1‐trifluoromethoxyphenyl‐3‐(1‐propionylpiperidin‐4‐yl) urea (TPPU) and the nuclear transcription factor kappa B (NF‐κB) pathway was inhibited by pyrrolidine dithiocarbamate and activated by phorbol‐12‐myristate‐13‐acetate in vivo and/or vitro, respectively. The inflammatory factors were detected by enzyme‐linked immunosorbent assay.

**Results:**

Hyperglycemia could exacerbate axonal injury, aggravate cell apoptosis and glial activation, worsen the loss of BBB integrity, increase the release of inflammatory factors, and upregulate the expression of sEH and NF‐κB. Inhibition of sEH could reverse all these damages and protect BBB integrity by upregulating the expression of tight junction proteins and downregulating the levels of inflammatory factors in vivo and vitro, while the agonist of NF‐κB pathway abrogated the protective effects of TPPU on BBB integrity in vitro.

**Conclusions:**

sEH was involved in mediating axonal injury induced by hyperglycemia after DAI by disrupting BBB integrity through inducing inflammation via the NF‐κB pathway.

## Graphical abstract

Hyperglycemia could exacerbate axonal injury, worsen the loss of blood‐brain barrier integrity, increase the release of inflammatory factors, and upregulate the expression of soluble epoxide hydrolase and nuclear transcription factor kappa B.

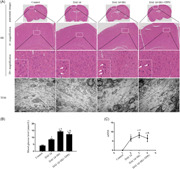

## 1 INTRODUCTION

Diffuse axonal injury (DAI), caused by traumatic brain injury (TBI) with acceleration or deceleration, is a microscopic damage of axons. The prognosis of several DAI patients is poor because of complications such as epilepsy, pulmonary infections, deep vein thrombosis, and hyperglycemia.^1^, ^2^, ^3^ It has been found that Glasgow Outcome Score in TBI patients was related to blood glucose levels.^4^ Hyperglycemia and hypoglycemia could both convey a poor prognosis. Compared to hyperglycemia caused by diabetes, hyperglycemia induced by stress could more easily lead to mortality in TBI patients.^5^ When TBI patients were hospitalized within the first 24 h, patients whose glucose level exceeded 160 mg/dL, resulted in poor outcomes regardless of the severity of injury.^6^ However, measures related to glycemic control such as intensive glycemic control could not be seem to improve the prognosis of DAI.

The cause of poor prognosis caused by hyperglycemia is partly related to rupture of the BBB.^7^, ^8^ Our previous study found that hyperglycemia could exacerbate BBB disruption via inflammation through the PPARγ/caveolin‐1/toll like receptor 4 pathway after DAI.^9^ However, the underlying mechanism by which hyperglycemia exacerbates brain damage and rupture of the BBB after DAI is still not clear. Soluble epoxide hydrolase (sEH) is an important enzyme that metabolizes bioactive epoxy fatty acids (EFAs) and plays a pro‐inflammatory role in central nervous system diseases.^10^ Genetic deletion of sEH and administration of sEH inhibitor could attenuate brain edema, apoptosis, inflammatory mediator, and ameliorate functional deficits post‐TBI. The expression of sEH can be induced by hyperglycemia.^11^ Inhibition of sEH activity provides significant protection against the detrimental effects of brain hippocampal microvascular inflammation induced by high dietary glycemia.^12^ The role of sEH in hyperglycemia‐induced axonal injury remains unknown after DAI.

In this study, the effects of an inhibitor of sEH on axonal injury, cell apoptosis and glial response after DAI combined with hyperglycemia were explored. The effect of sEH on BBB integrity was also studied in vivo and vitro exposed to high glucose (HG). We hypothesized that hyperglycemia after DAI led to elevated expression of sEH. The high level of sEH resulted in injurious differential protein expression changes characterized by axonal injury, cell apoptosis, glial response, inflammation, and BBB disruption. Furthermore, we aimed to determine whether the deleterious effects of sEH were related to the nuclear factor‐kappa B (NF‐κB) pathway in vivo and vitro.

## 2 EXPERIMENTAL PROCEDURES

### 2.1 Animals groups

Sprague‐Dawley (SD) rats were purchased from the Experimental Animal Center of Xi'an Jiaotong University (Experimental Animal Production License: SCXK [Shaanxi] 2006‐001). Rats were male, weighing 250–300 g and eight‐ to ten‐week‐old. Rats were acclimated for 1 week before the study procedures. Rats were fed a standard chow diet and housed in cages under 12 h light and dark conditions at 22 ± 0.5°C. All protocols were approved by the Animal Experimental Ethics Committee of Xi'an Jiaotong University. All experimental procedures were followed by the experimental animal operating procedures at Xi'an Jiaotong University.

A statistical power analysis was used to calculate sample size estimation. A one‐way analysis of variance (ANOVA) was used with the assumption that α = .05 and power = .80. By using a randomized digital table, 132 SD rats were divided into the following groups: control group (30 rats, 6 rats for each experimental project including pathology, transmission electron microscopy (TEM), western blot, enzyme‐linked immunosorbent assay (ELISA), Evans blue and water content), DAI 1d group (30 rats), DAI 1d+hyperglycemia (HG) group (30 rats), DAI 1d+HG + 1‐trifluoromethoxyphenyl‐3‐(1‐propionylpiperidin‐4‐yl) urea (TPPU, an sEH inhibitor) group (30 rats), DAI 1d+HG+pyrrolidine dithiocarbamate (PDTC) group (12 rats).^9^ Hyperglycemic rats were disposed of intraperitoneal injection of glucose (6.0 mL/kg; 50% glucose) at 0 and 12 h postinjury.^9^ To maintain the glucose levels >16.8 mmol/L within 24 h after injury, blood from tail vein were obtained at various times within 24 h postinjection (0, 12, and 24 h), and blood glucose concentrations were detected.^9^ TPPU (Sigma‐Aldrich) was intraperitoneally injected immediately and 12 h after DAI at the dose 1 mg/kg.^13^, ^14^ PDTC (Sigma‐Aldrich) was dissolved in saline. PDTC was intraperitoneally injected 100 mg/kg at 1 h before DAI and 50 mg/kg at 12 h after DAI.^15^, ^16^

### 2.2 Animal DAI model

Rat DAI model was established by a lateral head rotation device according to our previous studies. Rats were anesthetized with 1% (wt/vol) pentobarbital sodium (35 mg/kg).^17^ There are two lateral ear bars and an anterior teeth hole which were used to secure the rat head on the device. Rats were horizontally put onto the device, with its body at 30° oblique to the top of the laboratory table.^17^ After pulling the trigger, the rat head was rapidly rotated by 90° and suffered a sudden acceleration and deceleration. Rats in control group only underwent anesthesia.^17^ To perform the following examination, the rats were deeply anesthetized followed by perfusion with 40 g/L paraformaldehyde and/or normal saline. Six rats died after injury probably because of severe damage to the brainstem. These rats were excluded and replaced by new rats.

The specific region where we did the analysis in all experimental groups is dorsal cortex of frontal brain sections according to previous study.^18^

### 2.3 Hematoxylin and eosin (H&E) staining

Rat brains were collected, dehydrated, and embedded in paraffin. 6 µm thick slices was obtained from each section and was stained with H&E. Neuron morphology was observed by light microscope at 4× and 20× magnification.

### 2.4 Immunohistochemistry and semiquantitative analysis

Brain sections were cut, deparaffinized and rehydrated according to the experimental procedures mentioned above. Briefly, the antigen was retrieved in sodium citrate buffer. Endogenous peroxidase activity was quenched and bovine serum albumin was used for blocking. Primary antibodies, including β‐amyloid precursor protein (β‐APP) antibody (1:200, Abcam), glial fibrillary acidic protein (GFAP) antibody (1:400, Cell Signaling Technology [CST]), ionized calcium‐binding adapter molecule‐1 (Iba‐1) antibody (1:300, Wako), neurofilament light chain (NF‐L) antibody (1:200, CST), and neurofilament heavy chain (NF‐H) antibody (1:200, CST), were added to tissue samples and incubated overnight at 4°C. Secondary antibodies were used to incubate the samples. The slides were treated with streptoavidine and 3,3′‐diaminobenzidine. The slides were then dehydrated, mounted, and examined under a microscope. The expression of proteins was assessed by immunohistochemical scores which was determined by multiplying the quantity and staining intensity scores.^18^ The quantity scores range from 0 to 4 as follows: no staining, 0; 1%–10% of cells stained, 1; 11%–50%, 2; 51%–80%, 3; 81%–100%, 4. The staining intensity scores range from 0 to 3 as follows: 0, negative; 1, weak; 2, moderate; 3, strong.^9^ The total scores were ranged from 0 to 12. An experienced pathologist who was blinded to the experimental conditions was commissioned to detect the immunohistochemically stained sections.

### 2.5 Terminal deoxynucleotidyl transferase nick‐end labeling (TUNEL) assay

Apoptotic cells were determined by the DeadEnd Fluorometric TUNEL System (Promega) according to the manufacturer's protocols. Briefly, the slices were incubated with TUNEL reaction mixture for 1 h at 37℃ before diamidino‐2‐phenylindole nuclear staining. The TUNEL‐positive cells were counted in six fields which were randomly selected from the rat cortex under a fluorescence microscope.

### 2.6 Western blot analysis

Cortical samples were homogenized and centrifuged for 10 min at 4℃. An equal amount of protein (50 μg) was separated by sodium dodecyl sulfate polyacrylamide gel electrophoresis and transferred onto a polyvinylidene fluoride membrane. Then, the membrane was blocked for 30 min in nonfat dry milk buffer at 37℃ and incubated overnight at 4℃ with primary antibodies against claudin‐5 (1:1,000, Abcam), zona occludens 1 (ZO‐1) (1:1,000, Abcam), occludin‐1 (1:1,000, Abcam), NF‐κB (1:1,000, CST), sEH (1:1,000, Santa Cruz Biotechnology) and β‐actin (1:1,000, Abcam). The membranes were then incubated with horseradish peroxidase‐conjugated secondary antibodies for 1 h at room temperature. After that, bands were visualized. All data were normalized to the corresponding expression level of β‐actin using ImageJ 1.8.0.

### 2.7 Evaluation of BBB permeability by evans blue

The permeability of the BBB was evaluated at 1d after DAI. In brief, a 2% solution of Evans blue (5 mL/kg, Sigma‐Aldrich) was intravenously injected via the tail vein 1 h before sacrifice. Then, the rats were transcardially perfused with PBS under deep anesthesia. Brain tissues were removed, weighed, and homogenized in 50% trichloroacetic acid. Evan's blue concentration was measured by absorbance at 630 nm using a spectrophotometer.

### 2.8 Evaluation of brain edema

Brain water content (BWC) was measured by the wet‐dry method. Rats were killed at 1 day after DAI induction, and the brains were removed and weighed immediately after removal (wet weight) and kept in a 100℃ oven for 72 h to measure dry weight. BWC (%) was calculated by the following formula: [(wet weight−dry weight)/wet weight] × 100%.

### 2.9 Enzyme‐linked immunosorbent assay

Tissue was homogenized and centrifuged at 12 000 rpm and 4× for 15 min. The levels of tumor necrosis factor‐α (TNF‐α), interleukin‐ (IL‐) 1β, and IL‐6 were detected by ELISA kits according to the manufacturer's protocol. Briefly, inflammatory factor antibodies and the secondary antibody were applied in succession. Subsequently, the stop solution was added, and the optical density at 450 nm was measured. Data (pg protein) were normalized to mg of total protein.

### 2.10 Evaluation of neurological deficit

Neurological deficits at 1 day after DAI were assessed by modified neurological severity score (mNSS) tests according to a previously described protocol. All tests were performed by an investigator blinded to the experimental conditions and grouping. Briefly, the assessment for neurological deficit involves the motor, sense, balance, and reflex of rats. All the projects were scored with a range of 0‐18. Details are as follows: 0, no neurological deficit; 1–6, mild; 7–12, moderate; 13–17, severe; 18 losses of consciousness and death.

### 2.11 Transmission electron microscopy

The specific region where we did the analysis in all experimental groups was dorsal cortex of frontal brain sections as mentioned above, and was same as the region of HE staining. The dissected rat cortex tissues were fixed with 2.5% glutaraldehyde overnight, incubated with gadolinium tetroxide for 2 h, dehydrated with acetone and subsequently stained with uranium acetate. Then, ultrathin sections (60 nm) were made. All specimens were examined with an electron microscope (H‐7650).

### 2.12 Cell culture and treatment

Human brain microvascular endothelial cells (HBMEC) were cultured with medium which contained 10% fetal bovine serum. Cells were cultured in collagen coated transwells and placed in incubator which contained 5% CO_2_ and 95% air at 37℃ for four consecutive days. When the transendothelial electric resistance (TEER) was beyond 250 μΩ/cm^2^, a single layer of BBB was formed in vitro. TPPU was dissolved in culture medium at a final concentration of 50 μmol/L based on previous study.^19^

### 2.13 Oxygen‐glucose deprivation and groups in vitro

Cells were separated into five groups: control group, OGD 6 h group, OGD 6 h+ HG group, OGD 6 h+ HG + TPPU group and OGD 6h+ HG + TPPU+phorbol‐12‐myristate‐13‐acetate (PMA) group. In the control group, HBMECs were not treated with OGD. In the OGD groups, cells were treated with growth media without glucose and placed in a constant temperature at 37℃ environment filled with 5% CO_2_ and 95% N_2_ (vol/vol) for 6 h. For HG groups, cells were cultured on the inner surface of collagen‐coated Transwell inserts in complete culture containing 25 mM glucose.^20^ TPPU was added 30 min before OGD. In OGD 6 h+ HG + TPPU + PMA group, HBMEC were treated with PMA (0.1 µM) for 4 h before OGD according to previous study.^21^ Conditioned media and cells were collected for subsequent experiments.

### 2.14 TEER measurement

The protocol of TEER was in accord with the previous study. Cells were seeded into the upper chamber of the transwell. Then, the blank medium was added into the lower chamber. TEER was tested by a Millicell‐ERS instrument (Millipore) after incubating for various time intervals. The TEER values were measured in μΩ/cm^2^.

### 2.15 Horseradish peroxidase (HRP) flux

Culture medium containing the final concentration 0.5 mM HRP (SigmaAldrich) was added into the upper system. Then, culture medium was collected after incubation at 37℃ for 1 h. Colorimetric analysis were performed by using tetramethylbenzidine. After that, spectrophotometer was used to measured absorbance at 370 nm. The amount of HRP was quantified and assumed to be in nanograms per milliliter.

### 2.16 Statistical analysis

SPSS 18.0 (SPSS) was used to perform statistical analyses. Data are presented as the mean ± standard deviation (SD). Numerical data were analyzed by one‐way ANOVA to compare more than two groups, followed by LSD (L) to conduct a post hoc test. Significance was defined as *p*  <  .05. Shapiro–Wilk's method is recommended for the normality test. A normal distribution of all the data is shown.

## 3 RESULTS

### 3.1 Inhibition of sEH improved neurological outcomes and ameliorated axonal pathological injury in hyperglycemic rats after DAI

**Figure 1:**
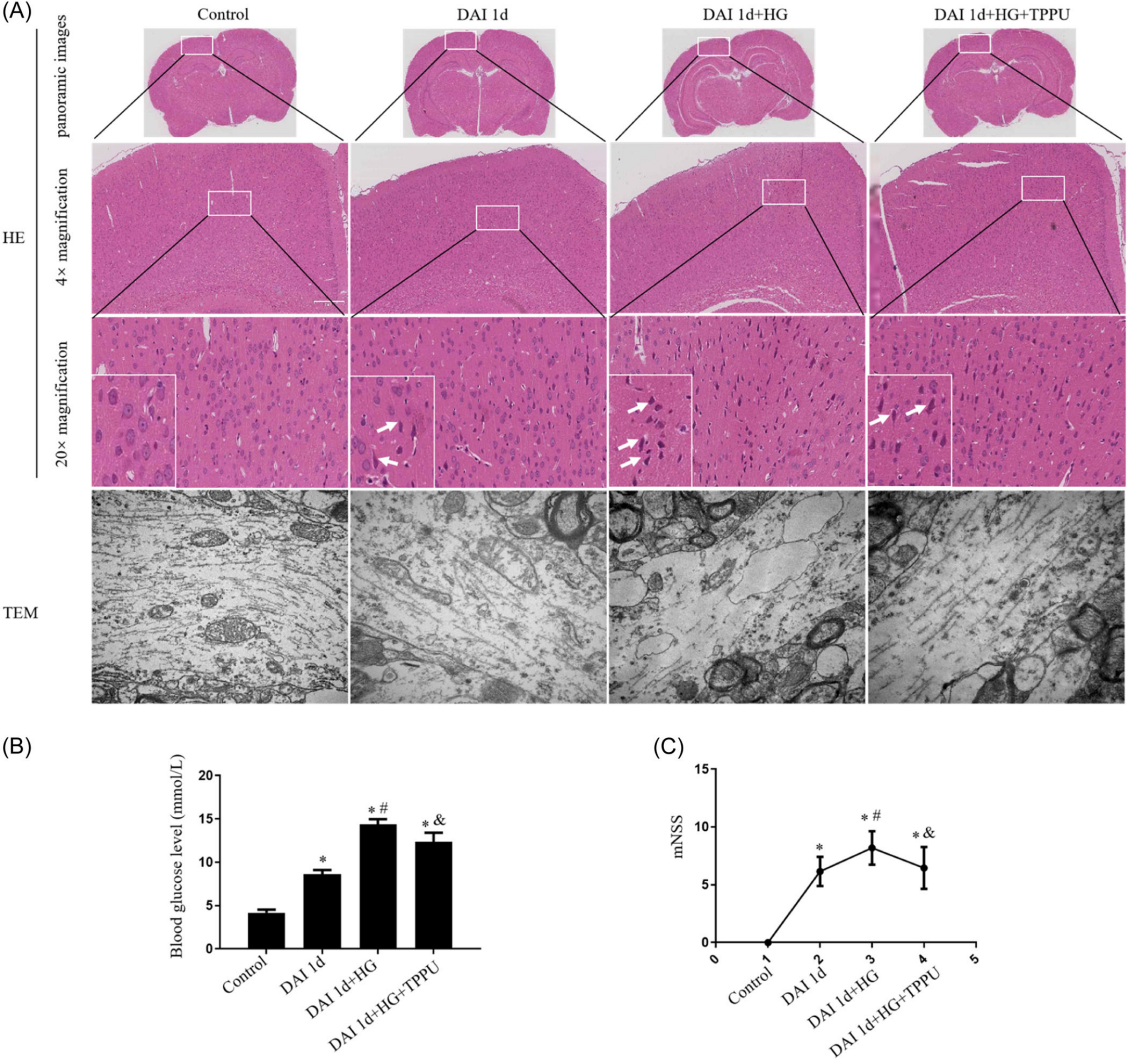
Pathological changes, glucose level and neurological outcome in rats suffering from hyperglycemia and sEH inhibitor after DAI. (A) Pathological changes were assessed by HE and TEM. A panoramic view of the rat brain and the highlighted area refers to the region where do the analysis in all experimental groups under different magnifications (4×magnification, 20× magnification). The small figures located in the bottom left corner were the magnification of particular sections. The white arrows indicated the pathological changes including neuronal pyknosis, swelling, and torsion neurons. For TEM, scale bar = 1 μm. (B) The graph showed the blood glucose level in each group. (C) The chart showed the mNSS scores for each group. *n* = 6; ^∗^ *p* < .05, versus the control group; ^#^ *p* < .05, versus the DAI 1d group; ^&^ *p* < .05, versus the DAI 1d + HG group.

In H&E sections, cell structures were integral in the control group. Neuronal pyknosis, swelling, and torsion neurons were observed in the cortex of the DAI 1d group. Compared to the DAI 1d group, these pathological damages were more serious in the DAI 1d + HG group. Compared to the DAI 1d + HG group, inhibition of sEH by TPPU relieved the abnormal histopathological changes in the DAI 1d + HG + TPPU group (Figure 1A). TEM results showed that in the control group, the structures of microtubules and mitochondria were consecutive and intact. In the DAI 1d group, the structure of microtubules was frayed, and the structure of mitochondria disappeared. Furthermore, compared to the DAI 1d group, the damage to microtubules and mitochondria was more serious in the DAI 1d+HG group. TPPU management displayed conspicuous partially preserved axon continuity in the DAI 1d + HG + TPPU group (Figure 1A).

Compared to control group, blood glucose level was significantly elevated in the DAI 1d group. Compared with the DAI 1d group, the blood glucose was higher in the DAI 1d + HG group. In comparison with the DAI 1d + HG group, TPPU could decrease the blood glucose levels (Figure 1B).

Compared with the control group, rats in the DAI 1d group exhibited higher mNSS scores, indicating neurological impairment induced by DAI. Compared to the DAI 1d group, the mNSS score was elevated in the DAI 1d + HG group, suggesting that hyperglycemia management worsened neurological impairment after DAI. Functional recovery was significantly increased in the DAI 1d + HG + TPPU group compared with the DAI 1d+ HG group (Figure 1C).

### 3.2 Role of sEH in axonal injury and glial response in the cortex of hyperglycemic rats after DAI

**Figure 2:**
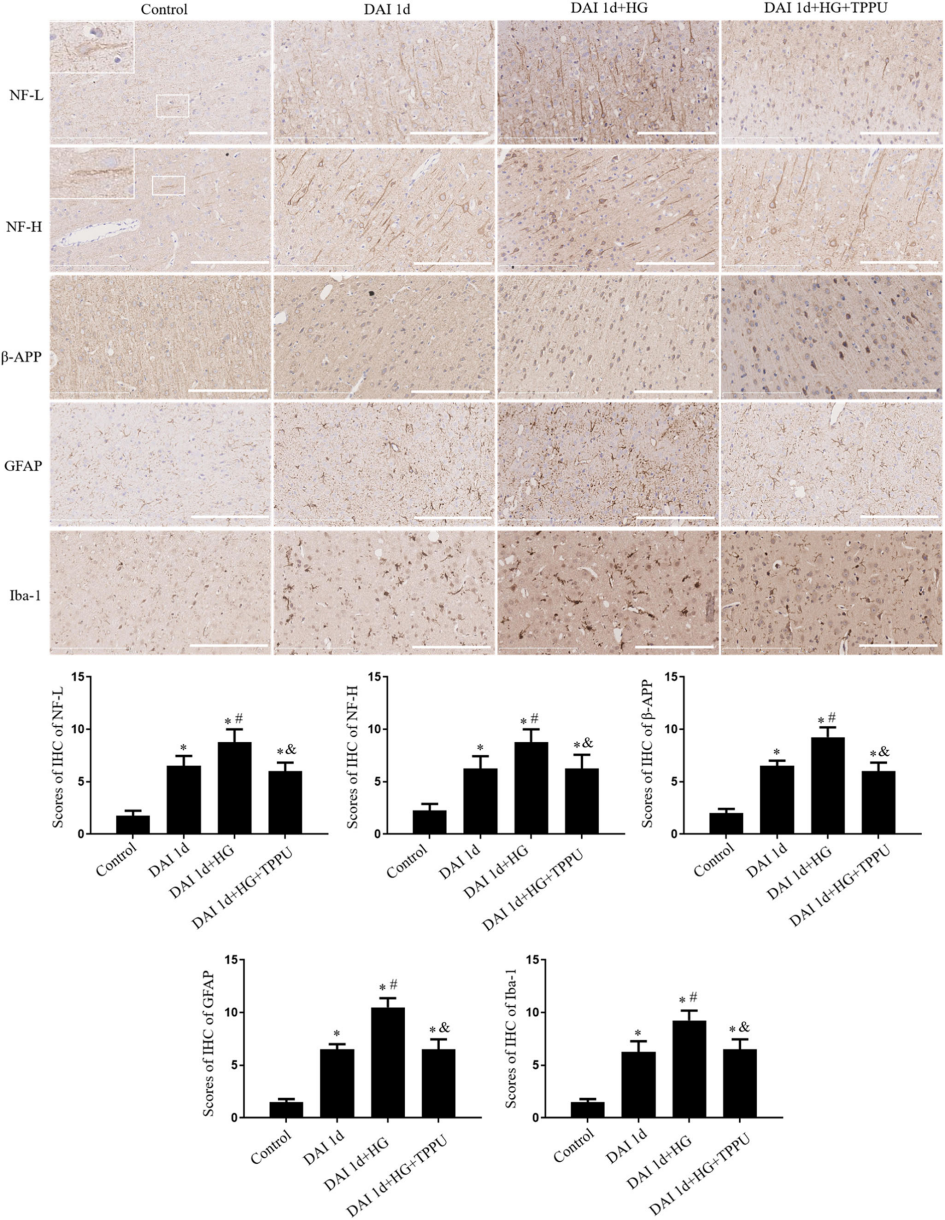
Effects of sEH inhibitor TPPU on axonal injury and glial response were assessed by the expression of NF‐L, NF‐H, β‐APP, Iba‐1, and GFAP through immunohistochemical staining (scale bar = 100 μm) after DAI. Enlarged image in the upper left corner of control group indicated the expression of neurofilaments under control conditions. *n* = 6; ^∗^ *p* < .05, versus the control group; ^#^ *p* < .05, versus the DAI 1d group; ^&^ *p* < .05, versus the DAI 1d + HG group.

NF‐L, NF‐H, and β‐APP can be used as markers of axonal damage, and Iba‐1 and GFAP can be considered as biomarkers of microglia and astrocyte, respectively. Compared to the control group, the expression of NF‐L, NF‐H, β‐APP, Iba‐1, and GFAP was higher in the cortex of the DAI 1d group. Compared to the control group, the expression of NF‐L, NF‐H, β‐APP, Iba‐1, and GFAP was further elevated in the DAI 1d + HG group. Compared with the DAI 1d + HG group, the expression of NF‐L, NF‐H, β‐APP, Iba‐1, and GFAP was decreased in the DAI 1d + HG + TPPU group (Figure 2).

### 3.3 Role of sEH in cell apoptosis in the cortex of hyperglycemic rats after DAI

**Figure 3:**
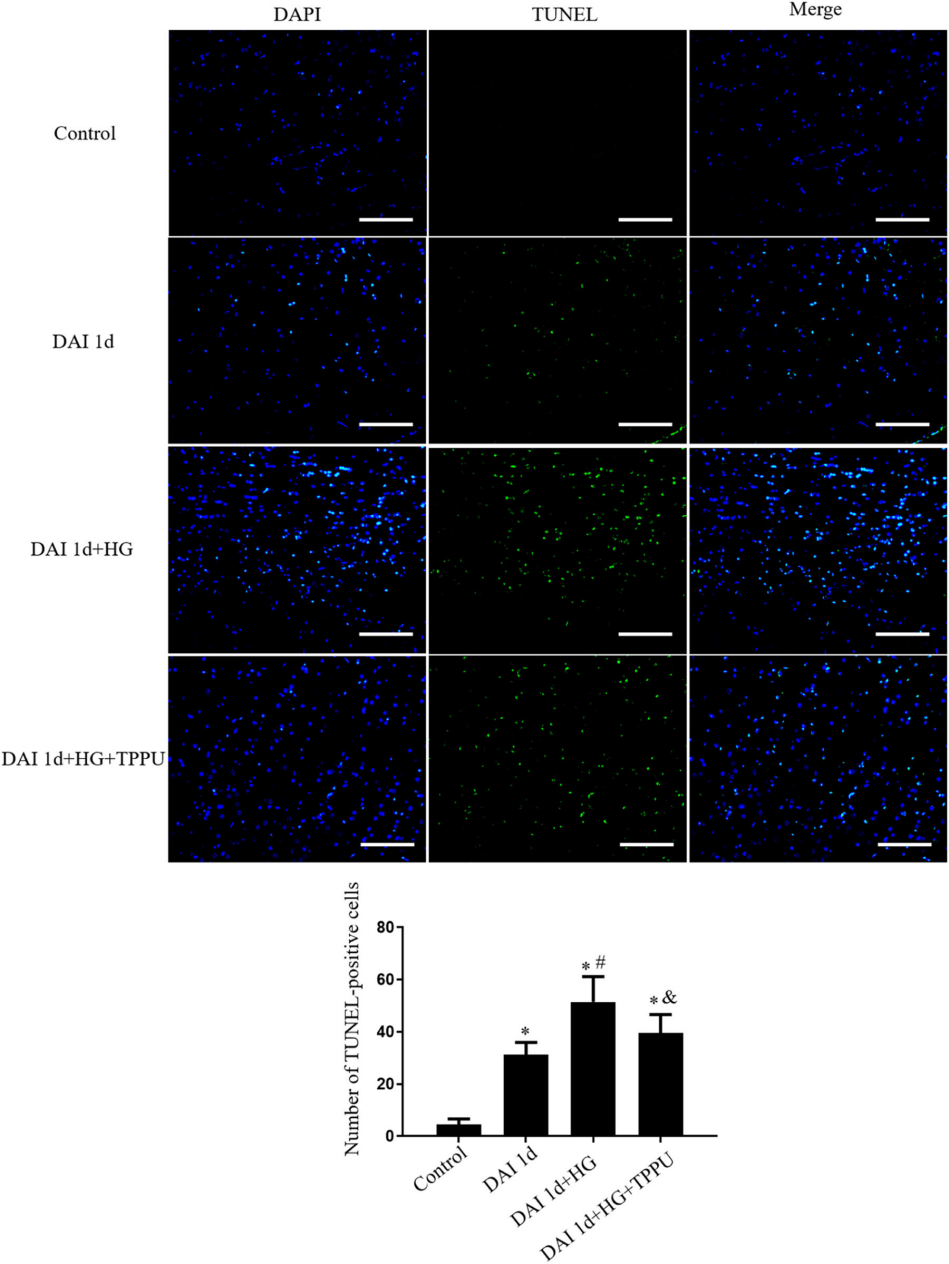
Role of sEH in cell apoptosis in hyperglycemic rats after DAI (scale bar = 100 μm). *n* = 6; ^∗^ *p* < .05, vs the control group; ^#^ *p* < .05, versus the DAI 1d group; ^&^ *p* < .05, vs the DAI 1d + HG group.

Cell apoptosis was assessed by TUNEL assay. In the control group, TUNEL‐positive cells were rarely detected. The number of TUNEL‐positive cells in the DAI 1d group was more than control group. Compared to the DAI 1d group, the number of TUNEL‐positive cells was increased in the DAI 1d + HG group, whereas TPPU treatment decreased the number of TUNEL‐positive cells in the DAI 1d + HG + TPPU group (Figure 3).

### 3.4 Roles of sEH in the regulation of BBB permeability in the cortex of hyperglycemic rats after DAI

**Figure 4:**
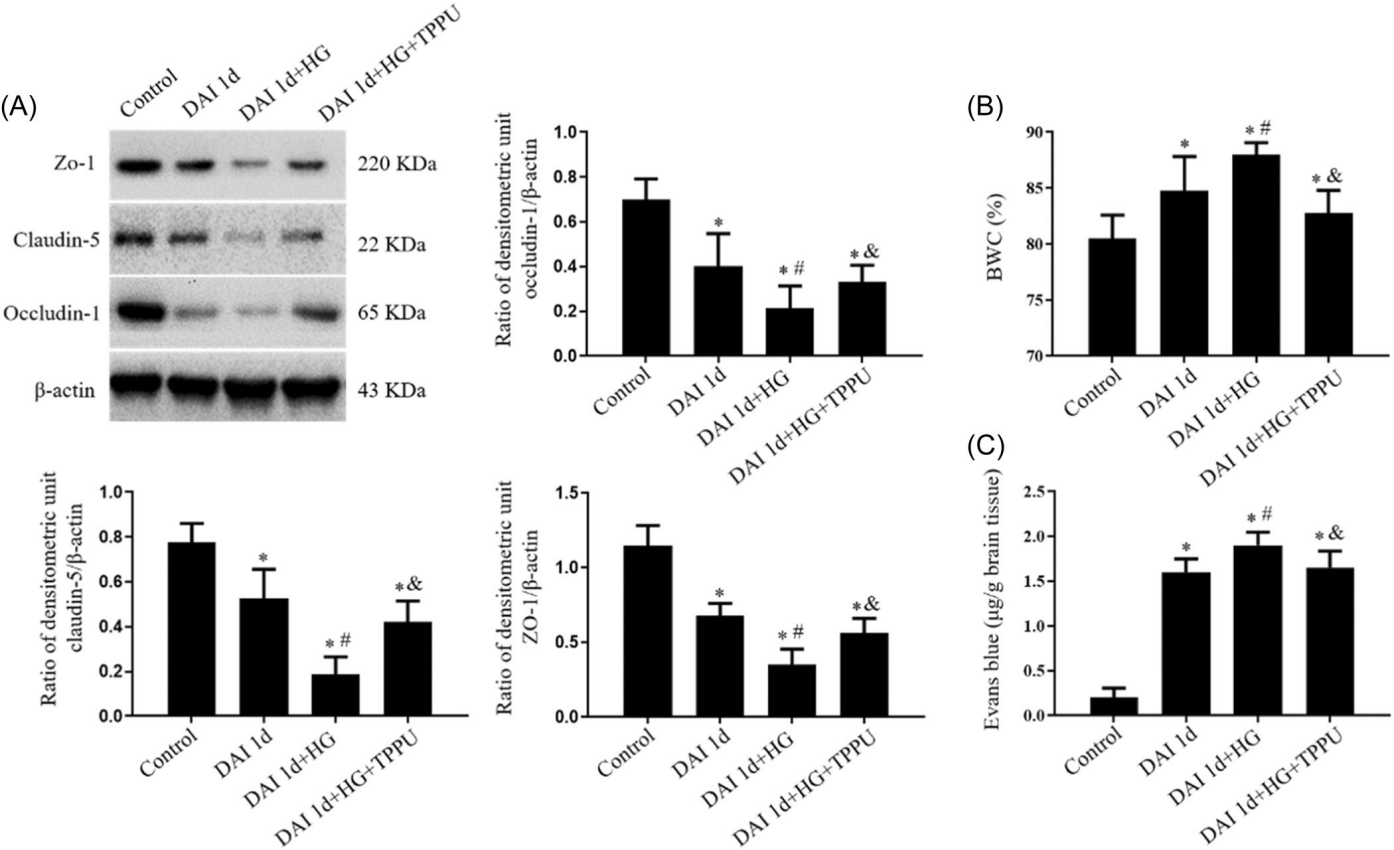
Roles of sEH in the regulation of BBB permeability in hyperglycemic rats after DAI. (A) Expression levels of claudin‐5, ZO‐1, and occludin‐1 were assessed by Western blot. (B, C) Evans blue diffusion and BWC were performed to evaluate the permeability of the BBB. *n* = 6; ^∗^ *p* < .05, versus the control group; ^#^ *p* < .05, versus the DAI 1d group; ^&^ *p* < .05, versus the DAI 1d + HG group.

The western blot results showed that the expression of claudin‐5, ZO‐1 and occludin‐1 was decreased following DAI. Compared to the DAI 1d group, hyperglycemia worsened the decrease in claudin‐5, ZO‐1, and occludin‐1 in the DAI 1d + HG group, indicating that hyperglycemia further disrupted the integrity of the BBB after DAI. TPPU treatment increased the expression of claudin‐5, ZO‐1 and occludin‐1 compared with the DAI 1d + HG group (Figure 4A). Compared with the control group, brain edema and leakage of Evans blue could be induced by DAI. Compared to the DAI 1d group, hyperglycemia treatment aggravated brain edema and leakage of Evans blue in the DAI 1d +HG group, whereas TPPU treatment decreased the BWC and Evans blue diffusion in the DAI 1d + HG + TPPU group (Figure 4B,C).

### 3.5 Inhibition of sEH decreased the level of inflammatory factors related to the NF‐κB pathway after DAI suffered hyperglycemia in vivo

**Figure 5:**
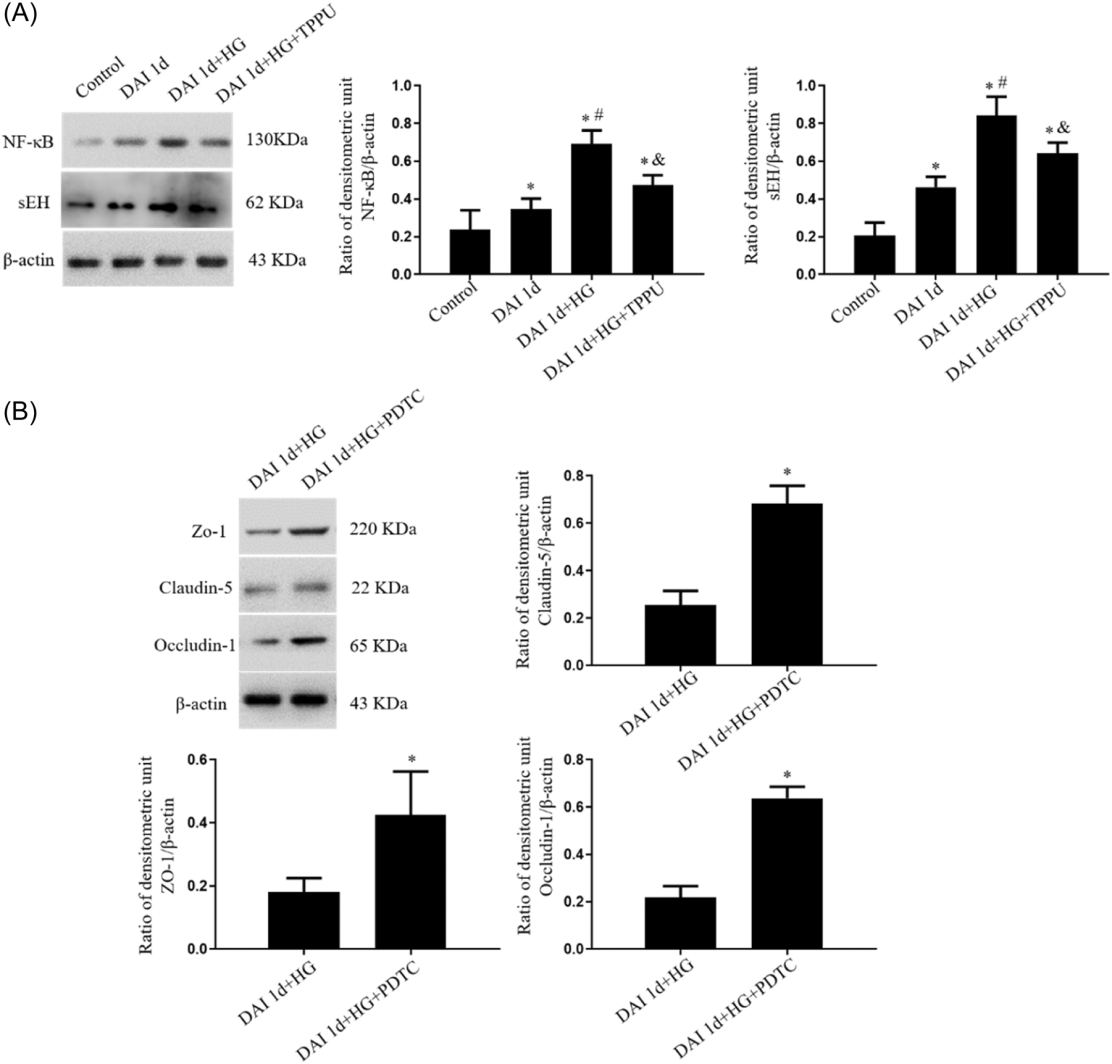
Inhibition of sEH decreased the expression of NF‐κB and inhibition of NF‐κB pathway protected the BBB after DAI under hyperglycemic conditions. (A) Expression levels of sEH and NF‐κB were assessed by Western blot. *n* = 6; ^∗^ *p* < .05, versus the control group; ^#^ *p* < .05, versus the DAI 1d group; ^&^ *p* < .05, versus the DAI 1d + HG group. (B) Expression levels of claudin‐5, ZO‐1, and occludin‐1 were assessed by Western blot. *n* = 6; ^∗^ *p* < .05, versus the DAI 1d+HG group.

The expression of sEH and NF‐κB in the DAI 1d group was higher than that in the control group. Compared to the DAI 1d group, sEH and NF‐κB expression was further increased in the DAI 1d+HG group. Inhibition of sEH by TPPU decreased the expression of sEH and NF‐κB in the DAI 1d+HG + TPPU group (Figure 5A). Compared with the DAI 1d+HG group, inhibition of NF‐κB by PDTC increased the expression of claudin‐5, ZO‐1, and occludin‐1 in the DAI 1d + HG + PDTC group, indicating that NF‐κB activation led to BBB disruption after DAI suffered hyperglycemia (Figure 5B).

**Figure 6:**
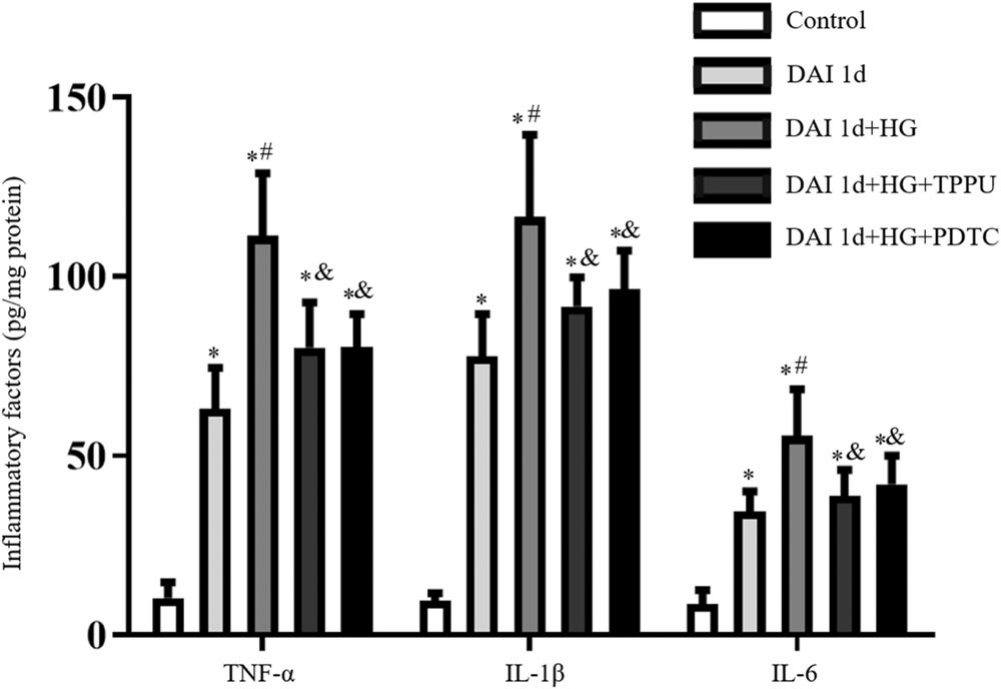
Inhibition of sEH and inhibition of NF‐κB decreased the level of pro‐inflammatory factors after DAI under hyperglycemic conditions, respectively. The effects of TPPU and PDTC on the levels of TNF‐α, IL‐1β, and IL‐6 in the rat cortex following DAI were determined by ELISA. *n* = 6; ^∗^ *p* < .05, versus the control group; ^#^ *p* < .05, versus the DAI 1d group; ^&^ *p* < .05, versus the DAI 1d + HG group.

The levels of pro‐inflammatory factors including TNF‐α, IL‐1β, and IL‐6 in the DAI 1d group were higher than that in the control group. Compared with the DAI 1d group, the levels of pro‐inflammatory factors were further increased in the DAI 1d + HG group. Compared to the DAI 1d+HG group, inhibition of sEH and NF‐κB both decreased the levels of pro‐inflammatory mediators in the DAI 1d + HG + TPPU group and DAI 1d + HG + PDTC group, respectively (Figure 6).

### 3.6 sEH disrupted of the integrity of the BBB through the NF‐κB pathway under hyperglycemic conditions in vitro

**Figure 7:**
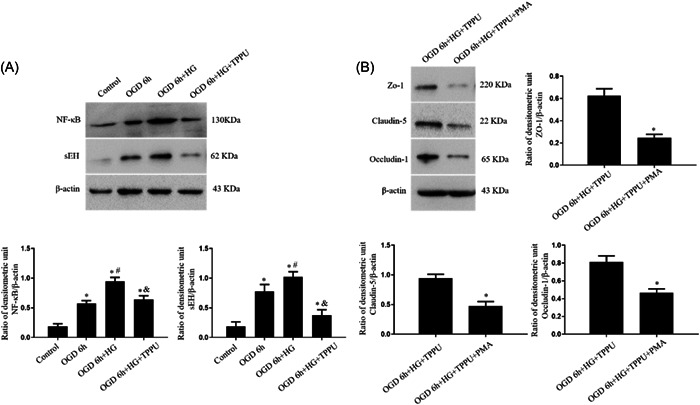
Inhibition of sEH decreased the expression of NF‐κB after OGD under hyperglycemic conditions and activation of NF‐κB pathway by PMA abrogated the protective effect on BBB model induced by TPPU after OGD under hyperglycemic conditions in vitro. (A) Expression levels of sEH and NF‐κB were assessed by Western blot. *n =* 6; ^∗^ *p* < .05, versus the control group; ^#^ *p* < .05, versus the OGD 6 h group; ^&^ *p* < .05, versus the OGD 6 h + HG group. (B) Expression levels of claudin‐5, ZO‐1, and occludin‐1 were assessed by Western blot. *n* = 6; ^∗^ *p* < .05, versus the OGD 6 h + HG + TPPU group.

In vitro, the expression of sEH and NF‐κB in the OGD 6 h group was higher than that in the control group. Compared to the OGD 6 h group, sEH and NF‐κB expression was further increased in the OGD 6 h + HG group. Compared to the OGD 6 h + HG group, treatment of TPPU decreased the expression of sEH and NF‐κB in the OGD 6 h + HG + TPPU group (Figure 7A).

Compared with OGD 6 h+HG + TPPU group, treatment of PMA (agonist of NF‐κB pathway) decreased the expression of claudin‐5, ZO‐1, and occludin‐1 in the OGD 6 h+HG + TPPU + PMA group (Figure 7B), indicating that PMA abrogated the protective effect of TPPU on tight junction proteins.

**Figure 8:**
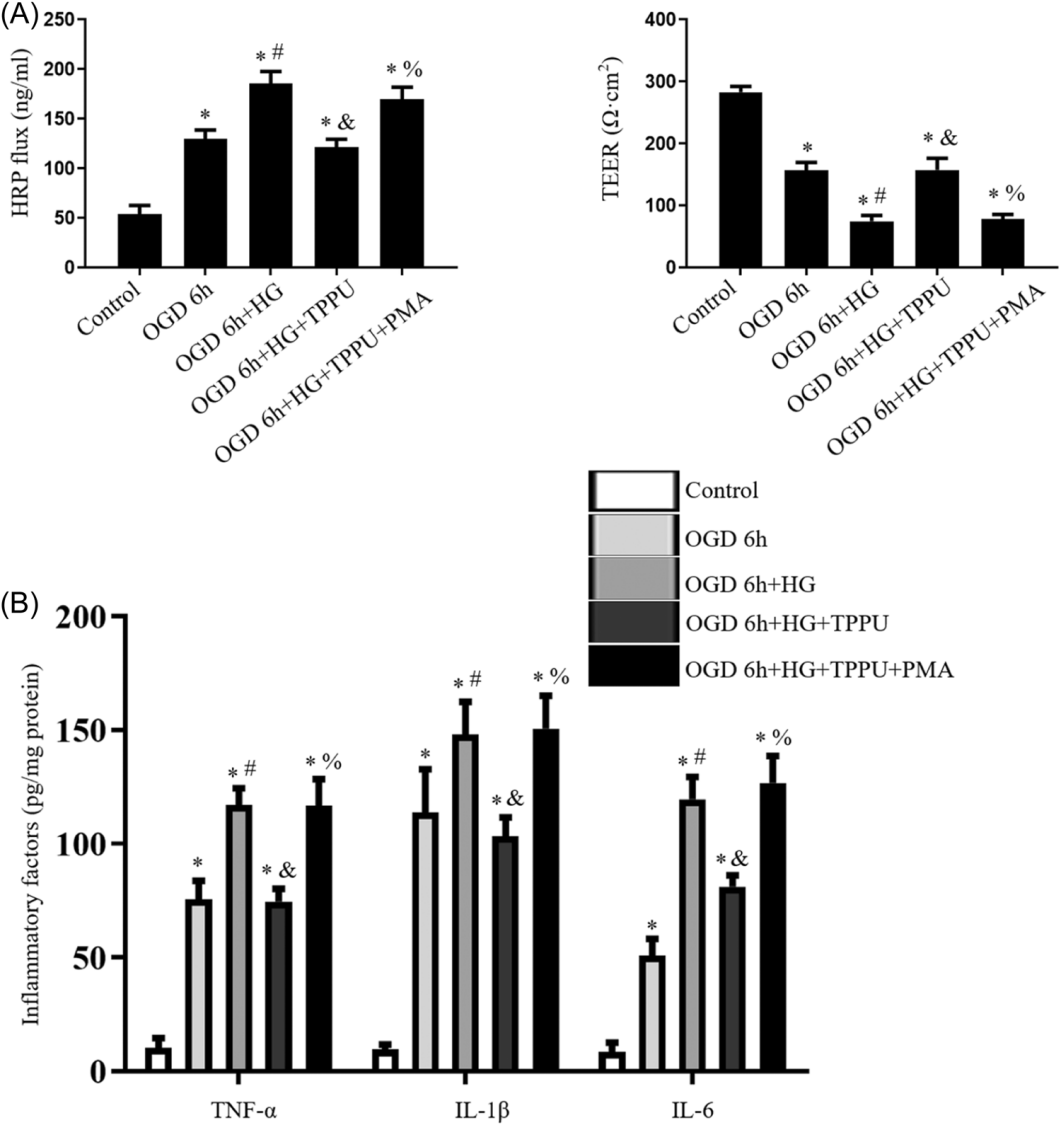
In vitro, inhibition of sEH protected of BBB permeability after OGD under hyperglycemic conditions by decreasing the levels of pro‐inflammatory factors through the NF‐κB pathway. (A) The permeability of BBB model in vitro was determined by HRP flux and TEER. (B) The levels of pro‐inflammatory cytokines after OGD under hyperglycemic conditions were assessed by ELISA. *n* = 6; ^∗^ *p* < .05, versus the control group; ^#^ *p* < .05, versus the OGD 6 h group; ^&^ *p* < .05, versus the OGD 6 h + HG group. ^%^ *p* < .05, versus the OGD 6 h + HG + TPPU group.

In vitro, the TEER in the OGD 6 h group was lower and the HRP flux was higher than those in the control group. Compared to the OGD 6 h group, the TEER was further decreased and the HRP flux was increased in the OGD 6 h + HG group. Compared with those in the OGD 6 h + HG group, TPPU treatment significantly increased TEER and decreased HRP flux in the OGD 6 h + HG + TPPU group. In the OGD 6 h + HG + TPPU + PMA group, PMA could reverse the changes of TEER and HRP flux compared to those in the OGD 6 h + HG + TPPU group (Figure 8A).

The levels of TNF‐α, IL‐1β, and IL‐6 in the OGD 6 h group was all higher than those in the control group. The levels of pro‐inflammatory mediators in the OGD 6 h + HG group were further increased than those in the OGD 6 h group. The levels of pro‐inflammatory mediators in the OGD 6 h + HG + TPPU group were lower than those in the OGD 6 h + HG group. In the OGD 6 h + HG + TPPU + PMA group, PMA could reverse the changes of levels of pro‐inflammatory mediators in the OGD 6 h + HG + TPPU group (Figure 8B).

## 4 DISCUSSION

Hyperglycemia has been regarded as one of the most used biomarkers with respect to the prognosis of various disorders, including TBI, cerebral ischemia/reperfusion, and subarachnoid hemorrhage.^22^, ^23^ Our previous study has found that hyperglycemia aggravates BBB disruption following DAI by increasing the levels of inflammatory mediators.^9^ However, the role of hyperglycemia in neuronal and BBB damage after DAI needs further research. In this study, we found that hyperglycemia exacerbated neurologic impairment and histopathological changes after DAI. Meanwhile, hyperglycemia generated axonal injury and aggravated TUNEL‐positive cells in the rat cortex after DAI. Hyperglycemia‐mediated axonal injury may contribute to the destruction of the BBB by increased inflammatory factors through the sEH/NF‐κB signaling pathway after DAI in vivo and vitro.

Axonal injury is one of the common early features of many central nervous system diseases and is associated with irreversible neurological dysfunction.^24^ The accumulation of β‐APP and overexpressed NF‐L and NF‐H have been considered signals of axon injury and indicated that the rapid transport of axons is interrupted.^25^ In this study, the expression of NF‐L, NF‐H and β‐APP and the number of TUNEL cells were higher after DAI, and hyperglycemia further elevated the expression of NF‐L, NF‐H, and β‐APP and the number of TUNEL cells. The results indicated that axonal injury and impaired neurological function was further worsened when rats were exposed to elevated glucose after DAI. In the previous study, hyperglycemia is also found to be one of the main factors associated to higher mortality when DAI was diagnosed.^26^ It was previously found that sEH deficiency contributed to significant alter of ambulatory movements and working spatial memory in the mouse.^27^ Moreover, deletion of sEH significantly attenuated functional deficits and protected BBB permeability after TBI.^11^ The level of sEH could be elevated after hyperglycemia treatment. The inhibition of sEH could downregulate genes involved in neurodegenerative diseases and endothelial cell functions to a greater extent with the high glycemic diet.^12^ In this study, hyperglycemia worsened neurological impairment after DAI in rats, while functional recovery was significantly increased by inhibiting sEH.

Prolonged hyperglycemia causes various microvascular changes and increases BBB permeability through the loss of tight junction integrity.^28^, ^29^ BBB disruption has been considered the initial step that induces neurological deficits and functional disabilities. Elevated endothelial permeability and decreased expression of occludin could be detected in HG‐challenged endothelial cells in vitro.^28^ Inhibition of sEH could also protect the BBB. In intracerebral hemorrhage mouse and ischemic injury rats, TPPU suppresses inflammation and secondary injuries and promotes functional recovery by alleviating BBB damage and increasing the regulation of microglial cell polarization.^19^, ^30^ sEH inhibition might also attenuate the progression of BBB injury in diabetic mice via AMPK/heme oxygenase‐1 (HO‐1) pathway activation.^31^ In this study, brain edema and BBB permeability could be induced by DAI and OGD and aggravated by hyperglycemia in vivo and vitro. TPPU treatment decreased the BWC and protected BBB integrity by increasing the expression of tight junction proteins.

Experimental study showed that TPPU could alleviate spatial learning and memory deficits in Aβ‐induced Alzheimer's disease mice through the regulation of glycogen synthase kinase 3β (GSK3β)‐mediated NF‐κB and Nrf2 signaling pathways.^32^ In an in vitro model of diabetic neuropathy, mouse schwann cells were incubated in HG medium for 48 h. After incubation, the expression and translocation of NF‐κB and the levels of IL‐6 and cell apoptosis were elevated.^33^ However, sEH inhibition can suppress the induction of apoptosis by exerting anti‐inflammatory effects through the NF‐κB pathway.^33^ In this study, inhibition of NF‐κB by PDTC reduced the extent of BBB damage by increasing the expression of tight junction proteins, in DAI rats suffering from hyperglycemia. In addition, inhibition of NF‐κB by PDTC and inhibition of sEH by TPPU both decreased the levels of pro‐inflammatory mediators. Treatment with agonist of NF‐κB pathway, PMA, could abrogate the protective effect of TPPU on tight junction proteins and levels of pro‐inflammatory mediators in the BBB models in vitro. Furthermore, DAI could be alleviated by protecting BBB integrity through decreasing the levels of pro‐inflammatory mediators in rats.^20^ All the results indicated that inhibition of sEH could protect BBB integrity and ameliorate neuroinflammation through the NF‐κB pathway after DAI with hyperglycemia.

## 5 CONCLUSIONS

All the results indicated that the level of sEH was elevated after DAI accompanied by hyperglycemia. The elevated sEH could aggravate axonal injury, and destroy BBB integrity by accelerating the release of pro‐inflammatory mediators through the NF‐κB pathways after DAI. Our findings provide evidence that sEH inhibitors could be a promising new way to treat DAI accompanied by hyperglycemia.

## AUTHOR CONTRIBUTIONS

**Xing Wei**: Data curation; Writing—original draft; Writing—review & editing. **Zhiguo Xing**: Data curation; Methodology. **Tingqin Huang**: Data curation; Formal analysis; Methodology. **Ming Zhang**: Methodology; Project administration; Software. **Jinning Song**: Project administration; Resources. **Yonglin Zhao**: Project administration; Writing—review & editing.

## CONFLICT OF INTEREST STATEMENT

The authors declare no conflict of interest.

## Data Availability

The data used to support the findings of this study are available from the corresponding author upon request.
